# Perceived Supportive Paradox After Diagnosing Human Papillomavirus: A Qualitative Content Analysis

**DOI:** 10.30476/ijcbnm.2021.88802.1547

**Published:** 2021-04

**Authors:** Narjes Nick, Camellia Torabizadeh, Mehdi Ghahartars

**Affiliations:** 1 Student Research Committee, Shiraz University of Medical Sciences, Shiraz, Iran; 2 Community Based Psychiatric Care Research Center, Department of Nursing, School of Nursing and Midwifery, Shiraz University of Medical Sciences, Shiraz, Iran; 3 Molecular Dermatology Research Center, Department of Dermatology, Shiraz University of Medical Sciences, Shiraz, Iran

**Keywords:** HPV Infection, Qualitative research, Social network, Support

## Abstract

**Background:**

Human papillomavirus (HPV) is the most globally-prevalent sexually-transmitted disease. Many stresses experienced by the patients after their disease is diagnosed affect the disease progression, and these problems and consequences demonstrate the importance of the support for the patients. The present research was conducted to explore the perception and experience of support in patients diagnosed with HPV.

**Methods:**

In this qualitative study, 24 participants (17 patients, 2 spouses of these patients, and 5 health service providers), selected using purposeful and snowballing sampling from April 2019 to March 2020, underwent an inductive content analysis conducted in dermatology clinic of Shahid Faghihi hospital, Shiraz, Iran. The data were collected through in-depth semi-structured interviews, all of which were recorded and transcribed. The data were analyzed in MAXQDA 2018 until data saturation was reached.

**Results:**

The patients aged 19-50 years old were married in 14 of the cases and their majority had genital or anal warts. Their level of education ranged from junior high school to an MSc degree. Perceived supportive paradox emerged as the main theme which consisted of 2 categories of supportiveness and lack of support.

**Conclusion:**

The present findings showed many challenges for the patients in the face of contradictory behaviors by their relatives and health service providers. Integrated systems are required to develop in order to promote the understanding of health service providers of HPV and counsel the patients to take appropriate strategies and, therefore, eliminate their confusion and reduce their anxiety.

## INTRODUCTION

Human papillomavirus (HPV) is globally considered one of the most prevalent sexually-transmitted diseases. Over 70% of sexually-active individuals are at the risk of infection with an HPV genotype. ^1^ HPV can cause cervical cancer as the fourth most prevalent cancer in females. A total of 570 thousand women were globally diagnosed with cervical cancer in 2018 and approximately 311 thousand of them have died from this disease. ^2^ HPV can cause anogenital cancers and warts in both genders. ^3^ In addition to the major impact of HPV infection in females, it is now well established that HPV can cause considerable disease in men at the genitals, anal canal, and oropharynx. ^4^ A meta-analysis in Iran reported the prevalence of HPV as 9.4% in healthy females and 77.4% in patients with cervical cancer. ^5 , 6^ In Iran, the heaviest burden of HPV was reported in 30-44 year-old individuals (51.8%), especially 30-32 year-old population. ^7^

In addition to the physical effects of HPV, its diagnosis can trigger different negative behaviors and feelings, including isolation, shocks, confusion, anger, powerlessness, self-blaming, stigma, and fear. ^8 , 9^ The psychological stress and its consequences, therefore, highlight the need for supporting the patients diagnosed with HPV. ^10^

Research suggests that better support provided by health service providers, family and friends promotes the health-associated quality of life. ^11 , 12^ Moreover, social network support is defined as various kinds of assistance one receives at the time of difficulties. This type of support can assist the individuals in the face of crises in managing and coping with their difficult life. ^13^ Research suggests the eagerness of females with HPV for emotional support and information by the time their disease is diagnosed. These patients should be individually provided with supportive and informative counseling services to mitigate their concerns after the initial disclosure of abnormal Pap smears. ^14 , 15^

Support has rarely been addressed in HPV patients despite its importance ^10^ and potential key role in preventing cervical cancer; ^16^ nevertheless, a large body of literature is devoted to quality of life in the patients, ^17^ the need of women for information and counselling services during HPV tests, ^18^ vaccination, ^19^ and the psychological effects of diagnosing the disease. ^20^ To the best of the authors’ knowledge, only one study that has qualitatively addressed so far the experience of support in female HPV patients in the US recommended the consideration of males in future studies. ^10^

The present study adopted a qualitative approach to manage the disease more effectively in the face of cultural, religious, and other contextual stigma attached to the disease. Qualitative research assists in understanding social phenomena from the perspective of the individuals involved rather than those of outsiders. ^21^ This study, therefore, sought to investigate the perception and experience of support in patients diagnosed with HPV.

## METHODS

The present qualitative study performed an inductive content analysis between April 2019 and March 2020 in the Dermatology Clinic of Shahid Faghihi Hospital, Shiraz, Iran as a referral center for HPV patients in southern Iran. The health service providers were also selected from their workplaces.

The participants were selected using purposeful and snowballing sampling, and maximum variation was observed in terms of sexual orientation, number of children, marital status, occupation, virus type, gender, age, duration of the disease, and level of education. The inclusion criteria were patients with HPV and willingness to participate in the study. The disease diagnosis was confirmed by a dermatologist or through virus typing tests. The only exclusion criterion was having cancer or other sexually transmitted diseases. 

The data were collected through face-to-face semi-structured interviews conducted by the first author in a comfortable location for the participants and mostly in a private room in the clinic. After recording the interviews using an audio-recorder, we transcribed them verbatim. 

The interviewer made field notes during all the interviews and documented all the relevant events in the clinic. Sampling and data collection continued until 21 participants were interviewed. Three more additional interviews were conducted to ensure data saturation although no new data was generated. Finally, 25 interviews lasting 30-100 minutes were conducted with 24 participants because the second participant was interviewed twice. The interviews began with general questions and continued according to the participants’ responses. Some questions included “How did your spouse or sexual partner react to your disease?”, “Could you please describe the behavior of health service providers on your visit and/or during consultation?”, and “How did this reaction affect you?”. Additional questions asked during the interviews for more clarification included “Could you give an example of this issue?”. The interviews asked the participants to talk about other issues they were willing to discuss or about questions not asked but they found it necessary to raise. An example of question asked from health service providers included “How do you explain the patient’s diagnosis to them?”

The data were simultaneously collected and analyzed. After listening to every interview for several times, we transcribed them in Microsoft Word and then imported them to MAXQDA 2018 to perform the data analysis. The texts were analyzed by employing conventional content analysis as an inductive approach proposed by Graneheim and Lundman. According to this method, the code scheme was directly extracted from the data. ^22^ The codes extracted after reading the texts word-for-word were reviewed and organized by their similarity to form the sub-subcategories. The analysis continued and similar subcategories were merged until the categories and the theme emerged. All authors did the data analysis and interpretation.

The Ethics Committee of Shiraz University of Medical Sciences, Shiraz, Iran approved the present study with the code of IR.SUMS.REC.1398.431. Before conducting the interviews, the participants were briefed on the study objectives and asked to sign written consent forms. The confidentially of their information and their right to withdraw from the study at their own discretion were also ensured. The researchers also ensured that the participants’ withdrawal at any time points would not result in consequences or negative effects on their medical care.

The evaluative criteria proposed by Lincoln and Guba were used to confirm the trustworthiness of the data. ^23^ Prolonged engagement, maximum variation, peer checking, and member checking were used to confirm the data credibility. For the sake of prolonged engagement, the researcher spent long hours in the field communicating with the patients. The experiences of the second and third authors with this method improved the data credibility. Observing maximum variation in selecting the participants enriched the variation in the study phenomenon. Transferability was also improved by performing the full description of the participants in terms of characteristics, culture, context and process of analysis; using purposive sampling; and observing maximum variation. The research team evaluated the process of coding and emergence of the categories and subcategories to achieve conformability. External auditing was performed to provide dependability.

## RESULTS

The 24 study participants aged 19-69 years included 17 patients and 2 of their spouses as well as 2 consultants and 3 physicians. Table 1 presents the demographic and disease-associated characteristics of the patients and Table 2 the demographic details of the health service providers.

**Table 1:** Demographic and disease-associated details of the patients

Participant number	Age (year)	Gender	Marital status	Education level	Number of children	Disease duration (Month)	Wart type	Virus type	Type of sexual intercourse	Sexual orientation
1	33	Male	Married	Associate’s degree	1	84	Genital	Type of wife’s virus: 18	Vaginal	Heterosexual
2	27	Male	Single	MSc	0	10	Genital	Unknown	Anal	Heterosexual
									Vaginal	
									Oral	
3	38	Male	Married	MSc	0	120	Genital	Unknown	Vaginal	Heterosexual
4	40	Female	Married	High school diploma	3	6	Anal	16,54	Anal	Heterosexual
									Vaginal	
									Oral	
5	34	Female	Married	High school diploma	2	8	Genital	45	Vaginal	Heterosexual
6	34	Female	Married	MSc	1	1	Genital	6	Anal	Heterosexual
									Vaginal	
									Oral	
7	19	Female	Single	High school diploma	0	1	Anal	Unknown	Anal	Heterosexual
8	50	Male	Married	Junior high school	2	21	Genital	Unknown	Anal	Heterosexual
									Vaginal	
									Oral	
9*	45	Female	Married	High school diploma	2		None		Vaginal	Heterosexual
10	27	Male	Single	BSs	0	36	Genital	Unknown	Anal	Heterosexual
									Vaginal	
									Oral	
11	24	Female	Single	Diploma	0	1 week	Anal	6,53,11	Anal	Heterosexual
12	43	Male	Married	High school	2	5	Genital	Unknown	Vaginal	Heterosexual
13*	38	Female	Married	High school	2		None		Vaginal	Heterosexual
14	28	Female	Married	Junior high school	2	2 weeks	Genital	Positive, not 16, 18	Anal	Heterosexual
									Vaginal	
15	32	Male	Married	BSc	0	12	Genital	11	Vaginal	Heterosexual
16	40	Female	Married	High school diploma	3	1	None	16	Vaginal	Heterosexual
									Anal	
17	30	Male	Single	High school diploma	0	10	Anal	Unknown	Anal	Bisexual
									Vaginal	
									Oral	
18	45	Male	Married	Junior high school	2	40 days	Genital	Type of wife’s virus: 6	vaginal	Heterosexual
19	45	Female	Married	Junior high school	2	4	Genital	6, 66	Anal	Heterosexual
									Vaginal	
									Oral	

* The patient wife currently has no symptoms of the disease and has not been tested, so her condition is unknown.

**Table 2:** Demographic information of the health service providers

Number	Gender	Age(year)	Occupational position	Work experience (year)	Education level
1	Female	39	Midwife- Counsellor	11	BSs
2	Female	37	psychologist-counselor	14	MSs
3	Female	69	Physician	45	OB & GYN, Gynecology Oncology
4	Male	50	Physician	25	general practitioner
5	Male	43	Physician	7	Dermatologist

The total number of codes emerging from the data were 450. According to Table 3, perceived supportive paradox emerged as the main theme with two categories, each of which including two subcategories. 

**Table 3:** Subcategories and categories of perceived supportive paradox as the main theme

Subcategory	Category	Main theme
Supportiveness of health service providers	Supportiveness	Perceived supportive paradox
Supportiveness of relatives		
Non-supportive social network	Lack of support	
Unprofessional behaviors of health service providers	

The majority of the participants found themselves in need of support when faced with their disease diagnosis. Confronting different individuals, however, caused them to face different and mostly contradictory experiences in a way that a patient concurrently experienced support and lack of support from relatives and health service providers. This supportive paradox is detailed as follows according to the patients’ experiences:

### Supportiveness

A number of participants reported the experience of receiving support. This category comprised two subcategories: supportiveness of health service providers and supportiveness of relatives. The sources of this supportiveness included family, friends, and health service providers. 

### a. Supportiveness of Health Service Providers

According to most of the participants, the medical team significantly affected their life by the way they treated them, established relationships with them and provided them with information about the disease. One of the participants said, *“I believe doctors are very influential. One’s morale is much affected by the doctor’s conduct”* (Participant 5).

Some of the participants reported receiving the necessary and true information about the disease from health service providers. The information they received were about sexual relationships, difference between viruses in type, using supplements to boost their immune system, treatment of warts, likelihood of clearance of viruses, and the interval between Pap smears. 

*“The doctor explained that this was a genital wart that is transmitted through sex as its only transmission route”* (Participant 12).

Certain information was sometimes distorted by the medical team. In fact, a strategy of the medical team was to prevent marital dissolution. Distracting the patient’s and spouse’s mind from the transmission routes of the disease and focusing the patient’s mind on abnormal transmission routes were often used to obliterate the suspicion of betrayal.

*“We seek not to disrupt their marital life. We tell them that sex is only one way of transmission; it may be a razor blade or dirty hands, so their minds are not preoccupied only with sex.”* (Participant 4, general practitioner)

Although this behavior of the health service providers often led to the continuation of the marital life of the patients, it caused negative consequences; for instance, claiming that the disease would not cause any problems by different doctors talking with the fiancé of participant 15 convincing her to get married; now, six months after the marriage, the couple wish they had never got married owing to the patient’s frigidity; nevertheless, they valued the doctors’ behavior in trivializing the disease for the woman.

*“The doctor told them not to ruin their lives for such a trivial matter …. and scolded my spouse for her intention to ruin her life, saying, *“get up, go and have your wedding; why did you postpone your wedding?. *“What the doctor told my wife then meant the world to me. It was not at all important to me what was going to happen afterwards …..”* (Participant 15)

On the other hand, raising the likelihood of transmission through ways other than sex by the health service providers caused the patients and their relatives to be obsessed with hygiene. The relatives, therefore, drew a line around and fenced off the patient. 

The participants found themselves worried by different causes, including examining the genital area, stigma of the disease, inappropriate treatments by the relatives, revengeful thoughts, anxious moments of receiving the typing results, concerns over the disease transmission to children and fear of human immunodeficiency viruses (HIV). The health service providers sometimes helped reduce this anxiety. One of the doctors said, *“I tell my patients not to worry as the virus often clears up. The virus certainly disappears at ages below 30 years, leaving no causes for concern.”* (Participant 3, OB &amp; GYN, Gynecology Oncology)

All the participants expressed their serious concern over their risk of developing cancer and wonder if all patients with HPV develop cancer and how long it takes for the cancer to be developed. The majority of the participants reported significant reductions in their anxiety by the medical team during this period and found soothing the patient important. 

*“The doctor boosted my morale a lot and told me not to fear, because there were no problems at all and he had seen thousands of these patients. I got a lot better and left his office very happily after he soothed me.”* (Participant 4)

Few of the participants reported that no discriminatory views in the health service providers caused positive effects.

### b. Supportiveness of Relatives

Supportiveness of relatives included the support provided by the patient’s family and friends. Some of the participants reported that the support provided by their relatives after their diagnosis included financial (procuring vaccine) and spiritual (prayers and vows for a brother or spouse) assistance, procuring herbal medicines, and sympathy with them.

*“Having no money, I used to borrow from my dad. I didn’t have any excuse then for taking money from him, so I decided to tell him the story. In short, my father bought me the vaccines.”* (Participant 10)

Some of the relatives sought to reduce the stress developed in the relationship between the patient and others. One of the participants described her sister’s reaction to the stress between she and her spouse:

*“My sister asked me to forgive my husband if possible. I asked her opinion about what to do and how to live with him. She asked me to forgive him for the mistake he had made.”* (Participant 19)

Some of the participants reported receiving support from their relatives, who arranged for an appointment to the doctor for them, accompanied them to health centers, followed them up for their treatment and vaccination appointments, and constantly asked after and talked to them. A friend of mine came with me and kept advising me against worrying and concerning myself with the result. According to the participants, their family members played a key role in easing their status by doing them a favour. Some of them had experienced kindness as *“empathizing”*, *“giving hope”* and *“sympathizing”* on the part of their family and friends. 

*“One of my sisters is the only person who is now empathizing with me. She calls and asks after me every day. She sympathizes with me.”* (Participant 18)

### Lack of Support

The category of lack of support comprised two subcategories of non-supportive social network and unprofessional behavior of health service providers.

### a. Non-supportive Social Network

Despite their urgent need for family support, patients may experience a serious sense of emotional void caused by worries associated with their disease diagnosis. Concerned over the disease stigma, some of the participants did not even seek their family support or experienced coldness in their family.

*“We are truly alone in life. Right now, there is no one around and this makes the problem worse.”* (Participant 3)

The lack of support and coldness of the family unit had led to a feeling of loneliness and helplessness and sometimes suicidal attempts.

*“There was no one to empathize with me. I wish there had been someone to whom I could disclose to calm me down, but there was nobody. I took 10 antidepressants and other tablets; somehow I just didn’t want to wake up in the morning or wanted to be in a suicidal state, so they would take me to hospital. I didn’t wish to die then, I just needed time to pour my heart out.”* (Participant 19)

After their disease diagnosis, the majority of the patients were scolded by their family members and friends. Many of the participants described their relatives’ behavior as rejecting and confining. Some of the participants identified emotional abandonment as a rejecting behavior of their relatives. The majority of the participants reported being sexually rejected by their spouse or sexual partner after their disease diagnosis. This rejection persisted for years after the diagnosis in the patients with the chronic disease. They found sexual intercourse, if any, to no longer be the same as before in quality. The spouse of one of the participants said, *“I wouldn’t let him touch me at all.”* (Participant 9)

One of the participants reported being rejected by his closest friend immediately after divulging information about his disease and that they never resumed their friendship. The spouse of one of the patients described her children’s treatment of their father after the diagnosis of his disease:

*“My son refused to shake hands with him because he was afraid the disease might be contagious. My husband kept saying that as if he had HIV, as if he had ….”* (Participant 13)

The confining behaviors of their relatives reported by some of the participants included preventing the mother from approaching her child and separating the patient’s things. About her husband’s behavior, one of the participants said, *“He told the children not to use the soap because it belonged to their mother.”* (Participant 16)

One of the participants was a single girl who reported being deprived by her family from social relationships and mass media following the disease diagnosis and disclosure of her extramarital relationships. Her family’s mistrust of her also caused her to be confined to home. 

Some of the participants reported the humiliating behavior of their family due to the disease stigma and contagiousness. 

*“I am constantly being insulted. They keep reminding me of my anal warts.”* (Participant 7)

The other experiences of the patients with the others’ reactions included the humiliating behavior of the spouse in front of the children, disclosing the disease without the patient’s permission, humiliating the patient’s pride, and bribing the spouse to attract their attention.

### b. Unprofessional Behavior of Health Service Providers

According to the participants, the way information was provided by the health service providers and their behavior could worsen the patient’s condition and affect their perception of the support received. Most of the participants reported receiving information only about infection with malignant carcinogenic viruses from some of the health service providers, who then did not explain about the percentage of cancer and the long cancer development process. The patients, therefore, feared that they might soon develop cancer. 

*“I got so scared when the doctor told me that this disease could develop into a cancer. I was convinced that I had cancer and it was all over.”* (Participant 11)

According to the participants, in addition to inducing cancer-related fear and frustration, the health service providers made non-scientific recommendations such as avoiding sexual intercourse, provoking concerns over transmitting the disease to children, stigmatizing, and discriminating against them, which increased their fear and stress. 

Desecrating and humiliating the patients in different wards of the treatment system associated with the disease stigma led to annoyance, frustration, violation of their privacy, and sometimes their withdrawal.

Describing the patients’ experiences with the behavior of the medical team, one of the counselors said, *“The patients were interrogated by the personnel about why they had developed this disease. One of my patients said that he had been blamed for the disease in many places he had been to. Many patients coming to the counselling room were shattered.”* (Participant 2, counselor)

Many of the patients experienced failed communication as aggressive, frank, and indifferent statement of negative information without considering the patient’s status. One of the patients whose wife was also a patient said, *“The doctor so explicitly and simply told my wife that she would have this wart throughout her life and that her uterus had to be ultimately removed; then, she throw herself out of the car on our way home.”* (Participant 18)

Some of the participants reported the failure of the health service providers to establish any relationships with the patients. The lack of information about the disease and its transmission routes and the risk of developing cancer caused the patients to ask the health service providers several questions. According to some of the participants, these questions remained unanswered even after the patients’ referral to the centers, and even the disease diagnosis was not discussed with them in some cases.

*“The doctor didn’t explain to me at all and didn’t even tell me the name of the disease. He immediately asked me to lie down on the bed and only checked my body from a distance.”* (Participant 12)

A few participants considered it a failure to provide the health system with information as a cause of the patients’ unawareness about their disease nature and their failure to observe preventive principles.

Although most of the participants had referred to different specialists, the treatment by the first doctor exerted the most significant and permanent effect on their mind. One of the participants reported being frightened by a doctor in his first visit and that he could never take his mind off this lasting fear. 

*“I could not take my mind off the first doctor’s words.”* (Participant 5)

Confirming this issue, the counselors found the counseling process of the patients with prior information more difficult. Despite making efforts to explain the truth about the disease, the patients still emphasized the issues raised by the first physician. In addition, these contradictions in the views of the medical team bewildered the patients and caused their distrust of the team and left their questions unanswered. 

*“Each one of them says something different. They don’t themselves know what they are doing. They have no correct information about this disease. I am bewildered and don’t know whose word to believe.”* (Participant 18)

## DISCUSSION

The present study examined the experiences of HPV patients with support. Analyzing the data extracted the theme of perceived supportive paradox consisting of two categories, namely supportiveness and lack of support. The participants received contradictory types of support from their family, friends and the health service providers. These contradictions can be explained by differences in the perspectives and cultural and family backgrounds of individuals.

In line with the present study, investigating the support provided by significant others, family, friends, and health professionals for female patients diagnosed with HPV in the US showed the beneficial effects of reappraisal, informative support, appointments and the reassurance offered. In contrast, the participants found the sympathy offered as support by relatives to alleviate their disease and its effects to be problematic, ^10^ whereas the present study participants considered the support by their families, friends, and health service providers helpful.

This discrepancy in results can be explained by differences in the cultural background; for instance, the challenging issue of informing cancer patients of their diagnosis and progression of their disease is still controversial in the Iranian culture and some are opposed to it. ^24^ Moreover, the present study participants reported being blamed, rejected, confined, and humiliated by their family, friends and health service providers and the failure of these three groups to properly communicate with them. In addition to cultural problems, the discrepancy can be explained by individual differences among the participants. As discussed earlier, the present study included both female and male patients as well as their spouses and health service providers.

The patients positive for HPV raise several questions about their disease. ^25^ On the other hand, and in contrast to HIV, given that the disease is covered up in Iran, the patients cannot access information sources approved by the Ministry of Health and Medical Education. Their sources of information are, therefore, limited to cyberspace that includes disintegrated contents published by different individuals. Communicating with patients and providing them with information are, therefore, crucial for addressing some of their concerns. ^14 , 25^ The patient-physician communication also plays a key role in patient training. ^26^ Some of the health service providers in the present study provided the participants with information, which is consistent with a previously-conducted study. ^10^ On the other hand, failing to appropriately communicate with the patients, the health workers provided them with no or incomplete information; for instance, the health service providers told the patients that HPV can cause cervical cancer without mentioning the high prevalence of the virus and its low risk of causing negative health effects on the vast majority of females exposed to it.

According to some of the participants, crowded offices and physicians’ irritability caused the communication failure and lack of information for the patients. Similarly, a meta-analysis in Iran showed the short duration of patient visits, which is inconsistent with the national guidelines that are based on global standards. Despite physician limitations with regards to this problem, ^27^ the effect of this problem on the patients’ life should not be overlooked; for instance, the present study found that the communication failure and incomplete information caused fear and bewilderment in the patients and increased their warts and the risk of the disease transmission. In contrast, lower stress levels were observed in the patients who received information from the health service providers.

The patients found both receiving information and the type of communication to be effective factors; for instance, some of the health service providers broke news about the risk of cancer or betrayal without sympathizing with the patients or ensuring the availability of support. Revealing the truth requires special skills and this can negatively affect the patients or change their life. ^26^ The communication of the health service providers with the study participants resulted in anxiety and frustration in some of the subjects. Their behavior also triggered tension between the couples in some cases, which is consistent with the results of a study suggesting poor communication skills in nurses in Iran. ^28^

Some of the health service providers distorted the information about the HPV transmission to save marital life of the patients. Although this strategy reduced tension between the couples, it developed obsession in the patients and their family. This white lie, as a supportive behavior, does not, therefore, appear to be an ethical way of protecting the couples’ relationship. Telling the truth is always expected of health service providers on the basis of the facts that disrespecting individuals’ autonomy is ethically incorrect and lying is wrong. ^29^

Diagnosing, evaluating, and treating HPV can provoke anxiety and significant negative psychosocial and psychosexual disturbance in all the patients. Health service providers should not only be kept scientifically up-to-date on HPV, but also provide information for the patients in a reassuring and supportive fashion. ^30^ In line with the literature, health service providers in the present study sympathized with them. ^10^ A meta-analysis showed that health service providers were expected to break the news to patients and their families in an empathizing manner to reduce their stress. ^31^

The stigma associated with HPV ^32 , 33^ can compromise the support provided for the patients. ^34^ Irrespective of their manifestation, stigma and discrimination may deprive the patients from the services they are supposed to receive from institutions to which they refer. ^35^ Females with HPV-induced cervical cancer may be unwilling to comply with the required follow-up procedures given their fear of the stigma of HPV. ^32^ Being negatively affected by the biased view and prejudice of the health service providers, as reported by some of the study patients, caused their stressful presentation and even their avoidance of presenting. The non-discriminatory behavior of the staff was found by a few of the participants to help correct their high-risk behaviors.

The stigma affected the behaviors of both health service providers and the patients’ families. Moreover, stress in the patients and their coping strategies were significantly affected by social network support, ^10^ which was provided by the family, friends and neighbors. ^36^

Compelling evidence suggests that family members constitute the main source of social network support. ^37^ The support discussed in the results section was provided for some of the participants by their friends and families. Research suggests the beneficial effects of family support in cancer ^38^ and HIV patients. ^39^ According to the present study participants, support by their families, especially their emotional support, played a key role in easing their conditions.

Some of the participants were, however, negatively treated by their friends and families. Research on HPV shows the difficulty of the female patients with accessing and locating sources of support owing to their geographical distance from their family members. ^10^ The stigma of HPV, as a sexually-transmitted disease, and concerns over its contagiousness caused the present study participants to experience negative reactions by their friends and families. 

Blame might be a driving force for stigmatizing of emotions and negative evaluation of character in women who suffer from cervical cancer caused by HPV. ^32^ Similarly, Chinese HIV patients were blamed and isolated or excluded by their families. ^40^ The present research found the stigmatizing attitude towards the HPV patients to relate to negative reactions, including blaming, isolating, humiliating, and rejecting, by the friends and families. Some of the study participants, therefore, expressed regret about seeking assistance of their friends and families. Religiosity is related to social acceptance and family in a country such as Iran in which religion plays a key role. ^41^ In addition to religion, the taboo of extramarital relationships made men avoid seeking their family’s support, as they feared the disclosure of their relationships and believed that raising them would not help solve their problem and further destroys the family’s emotional atmosphere, which can explain why most of married woman infected by their husbands were supported by their family, i.e. sister and mother, whereas single girls and women who had betrayed their husband were deprived of this support. Feeling betrayed and victimized and the disease contagiousness were reported by the women infected by their husbands as the reason why they sexually rejected their husbands.

According to the Iranian-Islamic culture, families and the community adopt conservative approaches to sexual issues and the related trainings and even the discussions. ^42^ Some of the participants identified traditionalism, religiosity, and rejection of extramarital relationships in their family as the causes of their deprivation of any types of sex and puberty education. Fearing negative reactions, they were also unwilling to divulge their disease to their family. Only one of the single boys reported being supported by his family owing to their high education level, awareness about the disease nature, and acceptance of extramarital relationships. On the other hand, the participants were humiliated, rejected, and isolated by their family and friends owing to their unawareness about the HPV transmission.

Receiving contradictory types of support by the patients exerted both positive and negative effects on their management of the disease. The present results that reflect the patients’ views on the behavior of other people can, therefore, be used in policy-making and planning for these patients.

The strengths of the present study included conducting interviews with both genders and the patients’ spouses and health service providers as well as taking a qualitative approach to determining the experience of the patients with the support they received after their diagnosis, which is rarely addressed in the literature. The study limitations also comprised failure to find homosexuals of either gender despite making great efforts to observe maximum variation in selecting the key participants and failure to interview with only bisexual participants. Another limitation of the study might be the gender difference between the female interviewer and male participants. This gender difference might affect the males’ responses to some questions.

## CONCLUSION

Developing an integrated system appears crucial for teaching health service providers and helping them better understand HPV and counseling patients to take appropriate strategies. One can facilitate it by giving similar information to patients, avoiding confusion, and eliminating unnecessary anxiety. Also, the lack of a service delivery system to adolescents constitutes a problem of the reproductive health system in Iran. The Iranian health system had better adapt sexual and puberty education for adolescents to the cultural context of the community. Future studies are recommended to investigate the effect of family-based educational intervention and counseling programs on the patients’ quality of life.
